# The Potential Role of Metalloproteinases in Neurogenesis in the Gerbil Hippocampus Following Global Forebrain Ischemia

**DOI:** 10.1371/journal.pone.0022465

**Published:** 2011-07-25

**Authors:** Luiza Wójcik-Stanaszek, Joanna Sypecka, Patrycja Szymczak, Malgorzata Ziemka-Nalecz, Michel Khrestchatisky, Santiago Rivera, Teresa Zalewska

**Affiliations:** 1 NeuroRepair Department, Mossakowski Medical Research Centre, Polish Academy of Sciences, Warsaw, Poland; 2 Neurobiologie des Interactions Cellulaires et Neurophysiopathologie (NICN), UMR 6184, CNRS, Aix-Marseille University, Marseille, France; Center for Regenerative Therapies Dresden, Germany

## Abstract

**Background:**

Matrix metalloproteinases (MMPs) have recently been considered to be involved in the neurogenic response of adult neural stem/progenitor cells. However, there is a lack of information showing direct association between the activation of MMPs and the development of neuronal progenitor cells involving proliferation and/or further differentiation in vulnerable (Cornus Ammoni-CA1) and resistant (dentate gyrus-DG) to ischemic injury areas of the brain hippocampus.

**Principal Findings:**

We showed that dynamics of MMPs activation in the dentate gyrus correlated closely with the rate of proliferation and differentiation of progenitor cells into mature neurons. In contrast, in the damaged CA1 pyramidal cells layer, despite the fact that some proliferating cells exhibited antigen specific characteristic of newborn neuronal cells, these did not attain maturity. This coincides with the low, near control-level, activity of MMPs. The above results are supported by our *in vitro* study showing that MMP inhibitors interfered with both the proliferation and differentiation of the human neural stem cell line derived from umbilical cord blood (HUCB-NSCs) toward the neuronal lineage.

**Conclusion:**

Taken together, the spatial and temporal profiles of MMPs activity suggest that these proteinases could be an important component in neurogenesis-associated processes in post-ischemic brain hippocampus.

## Introduction

Many recent studies have noted that ischemia resembles other brain injuries in producing enhanced neurogenesis in neuroproliferative regions of the adult rodent brain, including the subventricular zone (SVZ) of the lateral ventricles and the subgranular zone (SGZ) of the dentate gyrus (DG) of the hippocampus [1]–[4]. Ectopic neurogenesis has also been observed in degenerated hippocampal CA1 in animal models of global ischemia [5], [6].

The discovery of neurogenic responses subsequent to ischemic injury has led to the hypothesis that the expansion of the pool of endogenous progenitors could augment the regenerative capacity of the damaged areas. Therefore, the identification of mechanisms that promote the proliferation of progenitors, migration toward injured brain areas and differentiation into the phenotype of lost neuronal cells has become particularly relevant to the development of stem cell-based therapies.

It is hypothesized that following ischemic insult, neurogenesis proceeds as it does during embryonic development, involving the concerted action of cell surface and extracellular matrix molecules, thereby providing an environment which may be instructive or permissive to neurogenesis associated processes [7]. In this context, enzymes that modify the extracellular matrix and modulate both axonal guidance and cell adhesion molecules are particularly interesting [8].

The matrix metalloproteinases (MMPs) are one such group of proteinases known to play important roles in the ECM remodeling required for developmental processes.

MMPs belong to a family of secreted or membrane-bound endopeptidases, with 25 distinct mammalian gene products [9]. MMPs participate in numerous physiological and pathological processes through the processing of a variety of pericellular substrates including extracellular matrix proteins, cell surface receptors, cell adhesion molecules and growth factors [10], [11]. Whereas early up-regulation of MMPs, in particular gelatinases MMP-2 and MMP-9, has been mostly investigated in the context of their detrimental roles in brain ischemic injury [12], [13], their involvement in the neurogenic response of adult neural stem/progenitor cells in the ischemic brain has only been considered recently. MMPs are expressed abundantly in neural stem cells isolated from the human central nervous system (CNS) [14] and according to Mannello *et al.* [15] they have regulatory roles during the proliferation and differentiation of neural precursor cells in the embryonic mouse brain. Furthermore, Morris *et al.* [16] reported that mRNA expression of both MMP-9 and/or MMP-2 in neural progenitor cells of the SVZ increased several-fold after ischemic insult in adult rats. The report published by Lu *et al.* [17] showed that up-regulation of MMP-9 and MMP-2 in the SGZ of the dentate gyrus was compatible with the peak of post-ischemic neurogenesis in adult primate brains. It has been further proposed that MMP-9 facilitates neuroblast migration after ischemic stroke [18]–[20]. Altogether, these data strongly suggest the participation of MMPs in ischemic injury repair, favoring the migration of precursor stem cells from neurogenic into injured sites to replenish lost cells.

Despite ever-growing information concerning the involvement of MMPs in neurogenesis-associated processes *in vitro* and *ex vivo* in experimental stroke models, the proof of relevance *in vivo* after transient forebrain ischemia is still missing. Our previous study indicates that MMPs might indeed contribute to global ischemia-stimulated neurogenesis [21]. In the current work we further extend our investigation and evaluate whether the activation of MMPs in the brain hippocampus parallels the rate of neuronal progenitor cell proliferation and/or further differentiation after forebrain ischemia. In an effort to further elucidate the involvement of MMPs in neurogenesis-associated processes, we have also tested the effect of MMPs inhibitors on the development of a neural stem cell line derived from human umbilical cord blood (HUCB-NSCs). Our results show that dynamic evolution of MMPs activity matches the progression of proliferation and differentiation of stem/progenitor cells into mature neurons, highlighting the potential role of these extracellular proteinases in ischemia-induced neurogenesis.

## Materials and Methods

The following primary antibodies (source and final dilution) were used for tissue staining: rat polyclonal anti-BrdU (AbD Serotec, Raleigh, NC, 1∶200), mouse monoclonal anti-neuronal nuclear antigen (NeuN; Chemicon, Temecula, CA, 1∶500), mouse monoclonal anti-neurofilament 200 (NF-200, Sigma, Saint Louis, MO, 1∶500), and rabbit polyclonal anti-GFAP (DakoCytomation, Glostrup, Denmark, 1∶1000). Anti-rat FITC conjugated (Bethyl Lab, Montgomery, TX, 1∶1000), anti-mouse Alexa 546 (Invitrogen, Carlsbad, CA, 1∶500), and anti-rabbit Alexa 546 (Invitrogen, Carlsbad, CA, 1∶500) respectively, served as the secondary antibodies. For the immunocytochemistry of HUCB-NSCs, the primary antibodies utilized were: mouse monoclonal anti-Ki67 (Novocastra Lab Ltd., Newcastle,UK, 1∶100), mouse monoclonal anti-TuJ1 (Covance, Emeryville, CA, 1∶500), mouse monoclonal anti-MAP2 (Sigma, St Louis, MO, 1∶500), mouse monoclonal anti-galactocerebroside (GalC) (Chemicon, Temecula, CA, 1∶200) and anti S100beta (Swant, Bellinzona, Switzerland, 1∶1000). Secondary antibodies were conjugated to anti-mouse or anti-rabbit Alexa Fluor 546 (Invitrogen Molecular Probes, Eugene, OR, 1∶1000).

As mentioned in the Results section, some experiments were conducted with the following pharmacological agents added to the assay buffer or culture medium: broad-spectrum inhibitors of MMP – 1,10-O-phenanthroline (Merck, Whitehouse Station, NY,1 mM), and GM6001 (a peptidyl hydroxamate, Sigma-Aldrich Co, 25 µM), a non-selective MMPs inhibitor - doxycycline (Sigma-Aldrich, St Louis, MO, 60 µM ), competitive inhibitor of MMP-2 and MMP-9 SB-3CT [3-(4-phenoxyphenylsulfonyl)-propylthiirane, Sigma, 10 µM), an inhibitor of serine proteinases - Pefabloc SC [4-(2-Aminoethyl)-benzenesulfonyl fluoride, hydrochloride] (Roche Appl Sci, Mannheim, Germany, 5 mM) and the furin inhibitor Dec-RVKR-CMK (Calbiochem, San Diego, CA, 50 µM).

GM6001 was prepared as 1 mM stock solution and SB-3CT as 50 mM stock solution, each in DMSO. All the other agents were dissolved in PBS. For each agent, a corresponding diluting solution was used in these experiments as control.

### *In vivo* experimental design

#### Ischemic model

All experimental treatments were approved by the Local Commission of Ethics for Experiments on Animals.

Male Mongolian gerbils, weighing 50–70 g were used in the experiments. The animals were allowed free access to food and water. Forebrain ischemia was performed as described previously [22] by 5-minute bilateral ligation of the common carotid arteries under halothane/N_2_O anesthesia in strictly controlled normothermic conditions. Sham-operated gerbils served as controls. In all experiments six animals per time point and treatment were used.

#### BrdU labeling

5-bromo-2-deoxyuridine (BrdU; Sigma-Aldrich) dissolved in physiological saline was administered intraperitoneally (50 mg/kg per injection, in sterile 0.9% NaCl plus 0.007 N NaOH). Two-injection paradigms were used. In some experiments the animals received a single dose of BrdU and were sacrificed 24 h after the injection. This procedure was used to determine the number of cells that incorporated BrdU during a 24 h period at a specific time point after ischemia (6–28 days). In other experiments the animals received BrdU injections twice daily (12 h apart) for 3 consecutive days starting 6 days after the onset of ischemia. Animals in this group were sacrificed 14 and 28 days after the insult. This allowed us to determine the phenotype of the newborn cells. Sham-operated gerbils served as controls.

#### Tissue preparation and immunohistochemistry

At the scheduled time points, anesthetized animals were perfused transcardially first with phosphate buffered saline (PBS) followed by a fixative solution (4% paraformaldehyde, PFA, in 0.1 M phosphate buffer, pH 7.4). The brains were removed, and post-fixed for 3 h at 4°C in the same fixative solution. Following post fixation, brains were cryoprotected overnight in 20% sucrose solution (in 0.1 M PBS), frozen on dry ice and stored at −70°C. Double-labeled immunofluorescence was performed on free floating 25 µm coronal cryostat sections (seven to nine per animal) comprising the hippocampal formation of 6 animals per group.

For BrdU immunostaining, DNA was first denaturated in 2 N hydrochloric acid at 37°C for 60 min. Then tissue sections were incubated in 0.1 M sodium tetraborate (pH 8.5) for 15 min, blocked with 10% normal goat serum in PBS containing 0.25% Triton X-100 for 60 min, and incubated with anti-BrdU overnight at 4°C. Following the washing procedure, the primary antibodies were revealed by appropriate secondary anti-rat IgG2a FITC conjugated antibodies for 60 min at room temperature and in the dark.

The differentiation of BrdU-positive cells was monitored with mouse NF-200 and mouse NeuN as neuronal markers and GFAP as an astrocytic marker. After BrdU staining, the brain-tissue sections were incubated with primary antibodies overnight at 4°C. After being rinsed in PBS, the sections were exposed to secondary antibodies 1 h at room temperature. Negative controls were processed in the same manner on adjacent sections but with the primary antibodies omitted. Double labeling to determine the expression of phenotypic markers by BrdU expressing cells was verified using a confocal laser scanning microscope (LSM 510, Carl Zeiss, Jena, Germany). A helium-neon laser (543 nm) was utilized in the excitation of Alexa Fluor 546, while an argon laser (488) was applied in the excitation of FITC.

The number of BrdU-positive cells in the DG and CA1 area was assessed in an average of 7–9 coronal hippocampal sections per animal. To avoid double counting we did not analyze adjacent sections. Every section was evaluated using a computerized microscope system, and positive cells were displayed on a computer screen. All of the counting was performed under the fluorescence microscope and using a 20× objective.

#### *In situ* zymography

In order to localize activity of MMP-2 and MMP-9 within the brain hippocampus in control and ischemic animals and in HUCB-NSCs we conducted *in situ* zymography according to previously described methods [12], [23]. Thawed frozen, non-fixed coronal brain sections (25 µm thick) or HUCB-NSCs cultured on glass cover slips were incubated for 3 h at 37°C in a humid dark chamber in reaction buffer containing 50 µg/ml of FITC-labeled DQ-gelatin (Invitrogen Molecular Probes, Eugene, OR) that is quenched intramolecularly. Gelatin-FITC cleavage by tissue metalloproteinases (gelatinases) releases peptides whose fluorescence is representative of proteolytic activity. The sections were rinsed in PBS and fixed in cold 4% PFA for 20 min then mounted in fluorescent mounting medium (Dako) and observed using fluorescence microscopy. To confirm that the proteolytic activity is attributable to MMPs, some sections in each experiment were incubated in the above conditions with a broad spectrum inhibitor of metalloproteinases, 1 mM 1,10-O-phenanthroline. Fluorescence was visualized using an Axiovert 25 fluorescence microscope (Carl Zeiss, Jena, Germany) and confocal microscope. Images were captured on the Videotronic CCD-4230 camera, and processed by Axiovision image analysis system. All images subjected to direct comparisons were captured at the same exposure and digital gain settings to eliminate confounds of differential background intensity or false-positive fluorescent signals across sections.

In the next series of experiments double fluorescent labeling was performed in order to identify the cell types expressing gelatinolytic activity. After zymography, the sections were rinsed in PBS at pH 7.4, (3×10 min), preincubated in a blocking solution (10% goat serum+0.5% Triton X-100), and then incubated overnight at 4°C with proteins specific for either astrocytes (GFAP) or neurons (NF-200 and NeuN).

### Culture and treatments of HUCB-NSCs

HUCB-NSCs [24] were cultured as a mixed population of committed adherent progenitors and free-floating undifferentiated cells in F12/DMEM+2% FBS+ITS medium (Gibco) in stabilized conditions of 37°C and 5% CO_2_ in a fully humidified atmosphere. The pooled fractions of adherent and floating HUCB-NSCs were seeded at a density of 10^4^cells/cm^2^ onto fibronectin-coated glass plates (10 µg/ml in PBS). Prior to seeding fibronectin remained on culture dishes overnight at 4°C without air drying and the excess of substrate was then removed and plates rinsed with warm PBS. Following cell adhesion, the standard medium was replaced with the serum-free equivalent, either with or without MMPs inhibitor – SB-3CT, GM6001 or doxycycline, - at limiting concentrations. HUCB-NSCs cultures were left for 8 days *in vitro* (DIV) to grow and differentiate under the given conditions.

#### Culture growth

The same number of cells was plated on fibronectin-coated coverslips to estimate growth rate. Our observations found fibronectin to be a more effective ECM component in mediating cell growth and differentiation of HUCB-NSCs, as compared with laminin and collagen [25]. At the designated times of culture (4, or 8 DIV) in the presence or absence of MMP inhibitors, the standard medium was discarded and cells incubated (3 h at 37°C) with a medium containing Alamar Blue (Promega Corp.,Madison,WI). Fluorescence was read using a MultiScan Ascent FL (LabSystems Oy,Helsinki, Finland) spectrofluorimeter (by excitation wavelength 545 nm/emission 590 nm), and its level (proportional to the number of viable cells present on the multiwell plates) was converted to the number of surviving cells.

#### Immunocytochemistry

The cell cultures were fixed for 20 min with 4% PFA diluted in PBS. A blocking solution, containing 10% normal goat serum in PBS, was applied for 1 h at 25°C. The capacity of HUCB-NSCs to generate neurons and glia was examined through application of specific antibodies against neuronal (Tuj1, MAP2), oligodendroglial (GalC) and astrocytic (S100beta) antigens. Cell proliferation was evaluated using anti-Ki67 determining cells in the mitotic cycle. Immunoreaction with the primary antibodies was carried out overnight at 4°C. Cells were rinsed with PBS and then incubated for 1 h at RT with an appropriate secondary antibody conjugated to Alexa Fluor-546 (1∶1000, Molecular Probes). Controls for specificity of immunostaining were processed with either the primary or the secondary antibody excluded. Cell nuclei were visualized using 30 min incubation at RT with 5 µM Hoechst 33258 (Sigma). The labeled cells were examined under fluorescence and confocal microscope. Images were captured and processed as described above.

#### Analyses of HUCB-NSCs differentiation

Neurons, astrocytes and oligodendrocytes were counted manually. To quantify the percentages of cells expressing a specific marker in a given experiment, the number of immunoreactive cells in the whole population was determined, relative to the total number of Hoechst-positive non-apoptotic nuclei. In a typical experiment, 5000 cells per marker were counted and the results expressed as a percentage of total Hoechst-stained nuclei.

### Statistical analysis

All values are given as mean +/− SEM. Differences between means were also determined using one-way analysis of variance (ANOVA) followed by posthoc Bonferroni test. Statistical significance was deemed to be present if *p*<0.05.

## Results

### Time course of cell proliferation in the gerbil hippocampus after global ischemia

**Figure 1:**
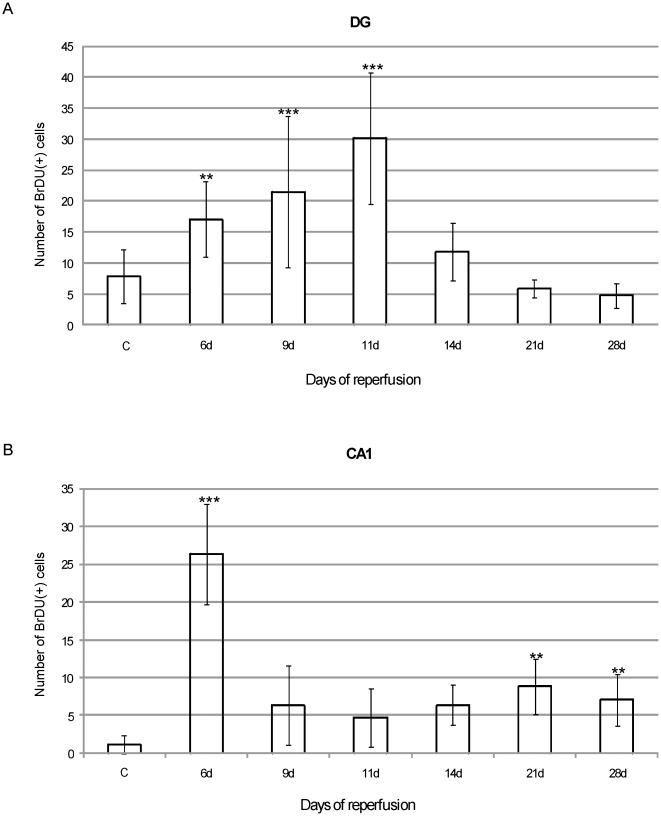
Time- course of cell proliferation in the adult gerbil hippocampus after global ischemia. Animals were subjected to 5 min of global forebrain ischemia followed by 6, 9, 11, 14, 21 or 28 days of reperfusion. BrdU was administered 24 h prior to sacrifice and brains were processed for BrdU immunohistochemistry. Graphs show the number of BrdU-labeled nuclei in the neurogenic SGZ of the DG (A) and in CA1 (B) in a sham operated control animal (C) and at different times after ischemia. The number of proliferating, BrdU-positive cells, increases markedly at 9–11 days of reperfusion in SGZ of the DG and at 6 days of reperfusion in CA1. Values represent the means ± SEM of six animals per time point. One-way ANOVA and Bonferroni test: **p<0.01 and ***p<0.001 indicate a statistically significant difference vs control value.

**Figure 2:**
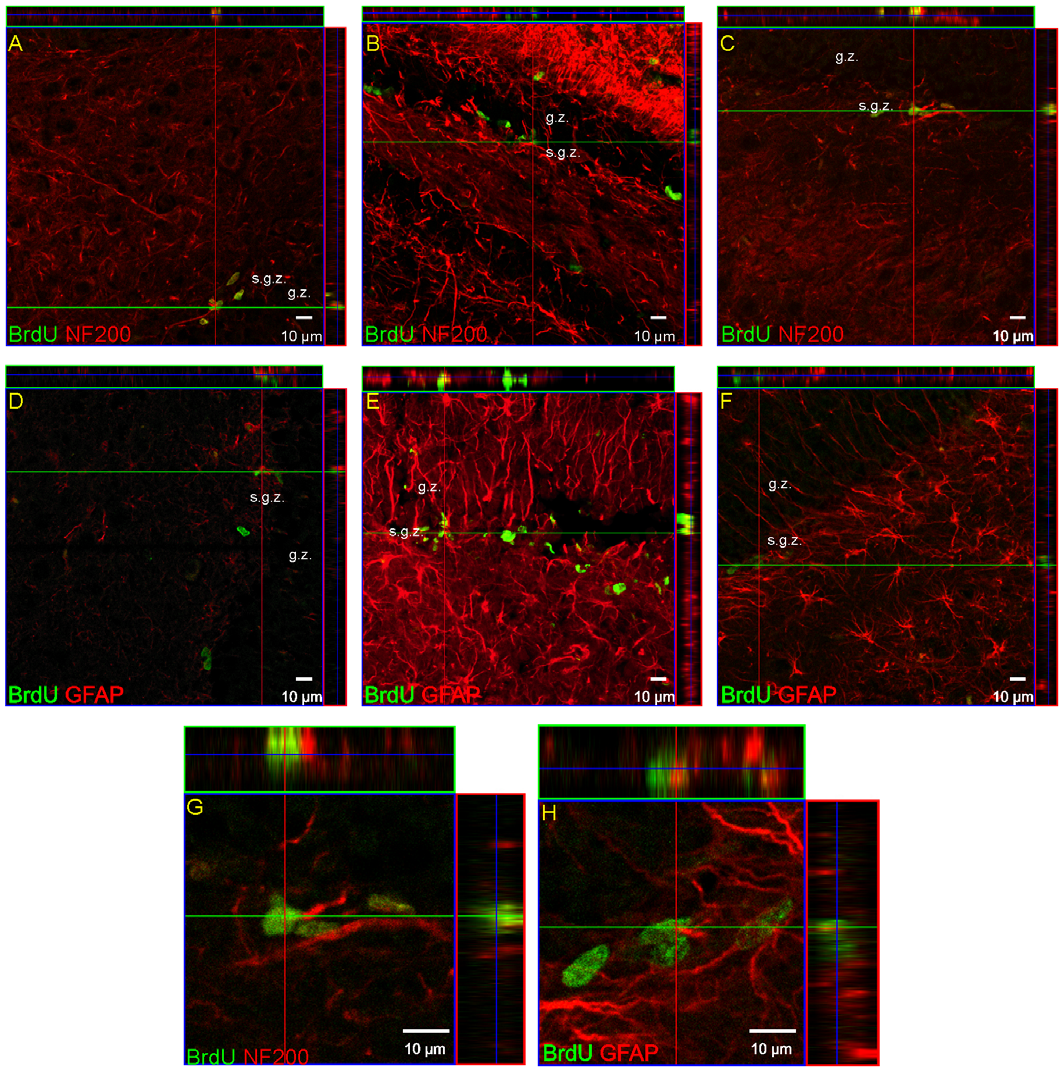
Newly-divided cells in the DG of adult gerbil hippocampus after global ischemia. Animals were subjected to 5 min of global forebrain ischemia followed by reperfusion. BrdU was administered 24 h prior to sacrifice. Brain sections were double-labeled with anti BrdU antibody (green) and anti-NF-200 (red) (A, B, C, G) or anti-GFAP (red) (D, E, F, H). Confocal photomicrographs show immunohistochemical reaction in control DG (A, D), 9 days after ischemia (B, E), and 28 days after ischemia (C, F). G, H represent magnification (z-stacks) of the picture C and F. Photomicrographs are representative of observations made from six animals per time point. Scale bar 10 µm. Abbreviations: s.g.z – subgranular zone, g.z. –granular zone.

**Figure 3:**
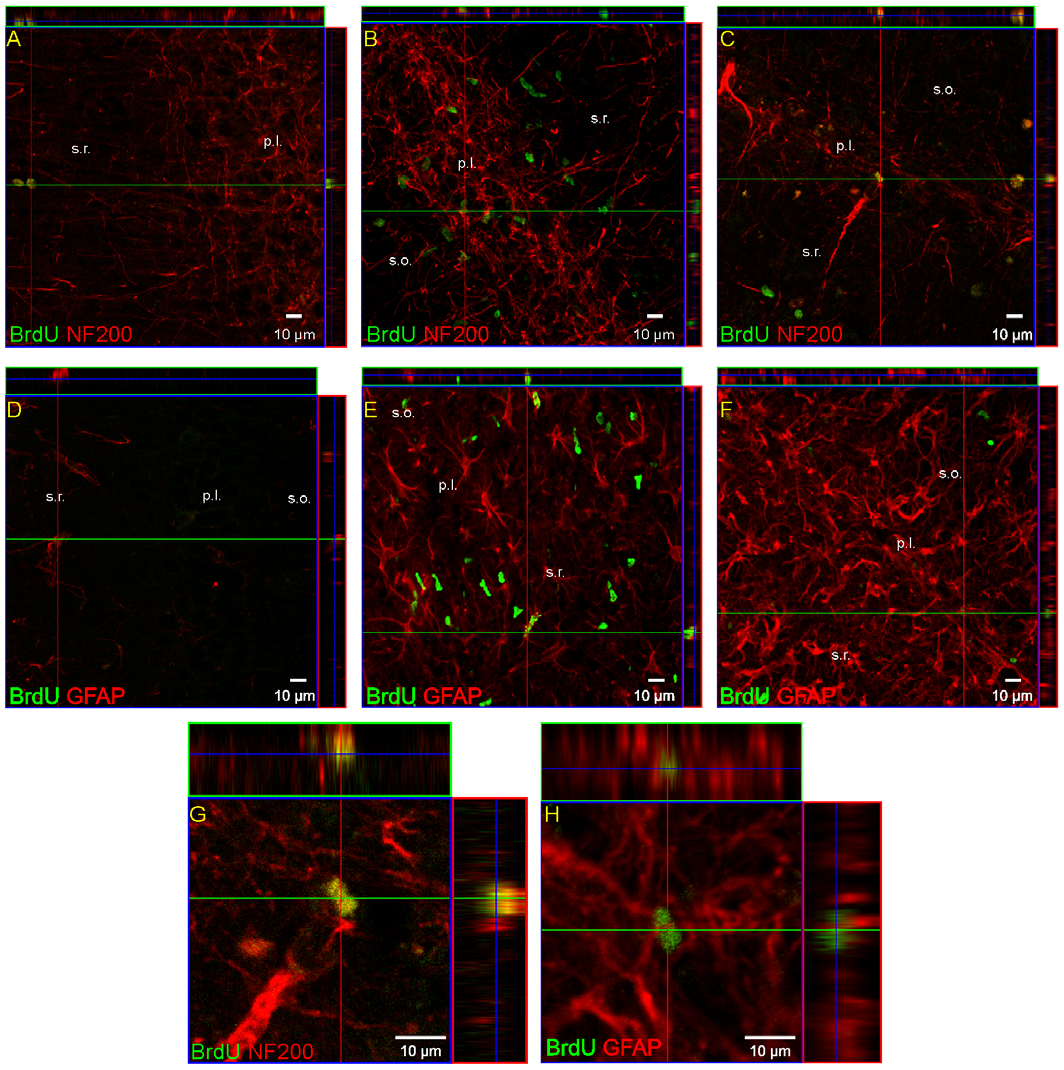
Neural identity of newly divided cells in the CA1 area after global ischemia. Animals were subjected to 5 min of global forebrain ischemia followed by reperfusion. BrdU was administered 24 h prior to sacrifice. Brain sections from a control animal (A, D) and from an animal 6 days (B, E ) and 28 days (C, F) after ischemia were stained for BrdU immunoreactivity (green) and neuron-specific NF-200 (A, B, C, G) or the astrocyte-specific GFAP (D, E, F, H) markers (red). C, H represent magnification (z-stacks) of the picture C and E. Six days after ischemia the higher than in control number of BrdU+ cells are seen within the damaged pyramidal cell. Intensive BrdU labeling was observed in *strata oriens* and *radiatum*. Depending on the CA1 area BrdU-positive cells expressed distinct antigens – NF-200 exclusively in pyramidal cell layer and GFAP in *strata oriens* and *radiatum* as well as in pyramidal cell layer. Photomicrographs are representative of observations made from six animals per time point. Scale bar 10 µm. Abbreviations: p.l. – pyramidal layer; s.o – *stratum oriens*; s.r.- *stratum radiatum*.

The time course of cell proliferation in the adult gerbil hippocampus was studied at specific time points after ischemic injury (6, 9, 11, 14, 21 or 28 days). For this purpose, animals received a single dose of BrdU 24 h prior to sacrifice. The number of newly generated cells was determined in the DG and CA1 structures by monitoring the incorporation and subsequent immunohistochemical detection of BrdU. A number of cells incorporating BrdU were found in the hippocampus of the normal, adult brain. After ischemia BrdU+ cell density increased markedly, though the labeling pattern revealed structure-dependent differences. In the DG the elevated presence of BrdU-positive cells was seen predominantly within the neurogenic SGZ, with the highest density (as compare to the sham control) observed between 9 and 11 days of reperfusion (Figs. 1A, 2B,E) [*F*(2, 75) = 16.04, *p*<.001]. Thereafter, cell proliferation appeared to return to the control level. Double labeling shows that some cells proliferating within 24 h in the SGZ express neuronal marker - NF-200 (Fig. 2A, B, C, G). GFAP-expressing cells were found occasionally among the BrdU-labeled population in the SGZ. Significant ischemia-induced elevation of the presence of BrdU+ cell nuclei within 24 h, as compared with the sham, was also observed throughout the CA1 region, with the greatest number of dividing cells present after 6 days of reperfusion [*F*(1, 51) = 30.48, *p*<.001]. The prolongation of recovery up to 28 days led to a reduction in the presence of immunoreactive cells, but numbers remained higher than in the sham animals [*F*(1, 49) = 7.90, *p* = .008] (Fig. 1B). As Fig. 3 demonstrates, the intensity of BrdU labeling is different depending on the topographical area. Only a few BrdU- positive cells were detected within the destroyed pyramidal cell layer, while the neighbouring structures, the *stratum oriens* and *stratum radiatum*, showed numerous cell nuclei with BrdU being incorporated. Importantly, these BrdU+cells expressed distinct antigens – NF-200 only in the layer vulnerable to ischemia, and GFAP in the entire CA1- in pyramidal layer as well as in *strata oriens* and *radiatum*.

### Phenotypic characterization of proliferating cells after hypoxia/ischemia

**Figure 4:**
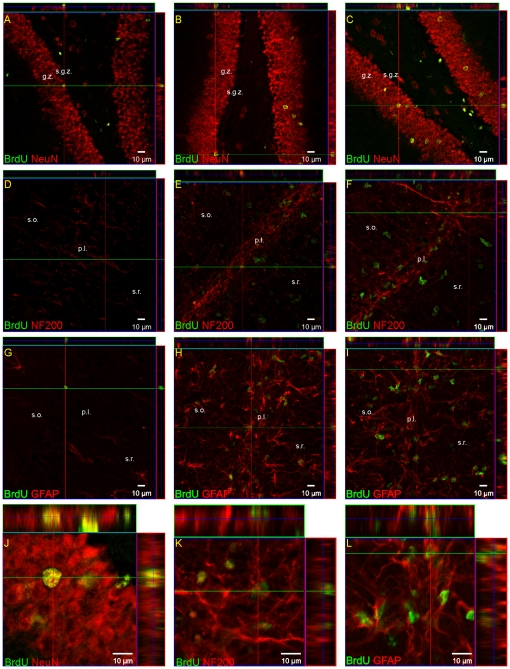
Neurogenesis in the adult gerbil hippocampus after global ischemia. Animals were subjected to 5 min of global forebrain ischemia followed by reperfusion. BrdU was administered twice daily 6–9 days after ischemia. Brain sections from a control animal (A, D, G), and from an animal 14 days (B, E, H) or 28 days (C, F, I, K, L) after ischemia were stained for BrdU immunoreactivity (green), for specific neuronal markers (NF-200 and NeuN -red) and astrocytic marker (GFAP-red) in the DG (A, B, C) and the CA1 (D–I). J, K, L represent magnification (z –stacks) of the picture C, F, I, respectively. Note co-localization of BrdU with mature NeuN-positive neurons in SGZ, SG and hilus at 14 days after ischemia (B) and increased number of BrdU/NeuN positive cells in SG at 28 days after ischemia (C, J). Photomicrographs are representative of observations made from six animals per time point. Scale bar 10 µm. Abbreviations: s.g.z - subgranular zone, g.z.- granular zone, h - hilus, p.l.- pyramidal layer.

To further characterize the fate of cells incorporating BrdU, sections from sham and ischemic gerbils were double-stained for BrdU and known neuronal antigens - NF-200 (detected in immature as well as in mature neurons) and NeuN (for mature neuronal cells) and GFAP for astrocytes. For the purpose of these studies, animals received multiple BrdU injections on days 6–9 after ischemia, and were sacrificed 14 and 28 days of reperfusion. Representative images are shown in Fig. 4. Striking proliferation of the precursor population was observed during the investigated period of recovery following ischemia in the DG with the most pronounced expansion of BrdU-positive cells at 4 weeks (Fig. 4C, J). Numerous BrdU-positive nuclei in this area were closely associated with mature neurons expressing NeuN, though some showed positive immunoreaction with NF-200 (see Fig. 2), a marker of neurofilaments.

BrdU/NeuN labeled cells were also observed occasionally in the hilus. At the time of recovery, particularly at 28 days, numerous BrdU/NeuN-positive, newly-generated cells, integrated the granular cell layer, suggesting the occurrence of short-distance migration (Fig. 4C). Some (albeit significant fewer) double-stained cells of the BrdU/NF-200 and BrdU/NeuN types also appeared in the SGZ of control animals showing the basal level of cell differentiation.

During the investigated time of reperfusion the numbers of BrdU-positive cells in the damaged CA1 pyramidal cell layer outnumbered those in the control. Some of them, sparsely distributed within this structure, showed double labeling with BrdU and NF200, which was better manifested at 28 days of recovery (Fig. 4D, E, F). We did not observe co-localization with NeuN, the marker specific of developed neurons. An increasing number of BrdU-positive cells were observed in the structures neighboring the pyramidal CA1 layer. However, these expressed the GFAP antigen and showed morphology characteristic of astrocytes (Fig. 4G,H,I).

### The effect of ischemia/reperfusion on the activity of MMPs in the hippocampus

**Figure 5:**
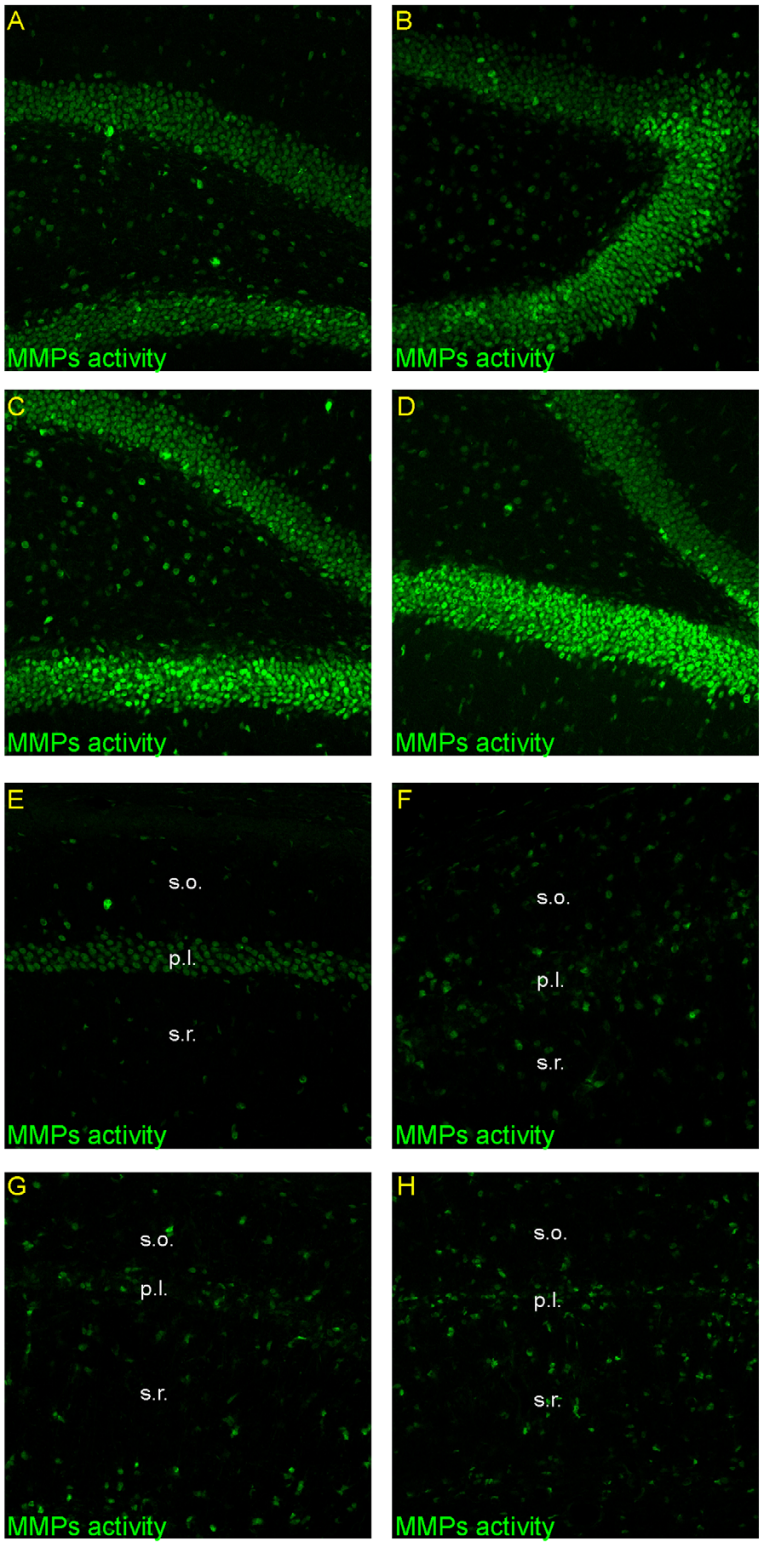
Activity of metalloproteinases in the adult gerbil hippocampus after global ischemia. Animals were subjected to 5 min of global forebrain ischemia followed by reperfusion. Confocal photomicrographs showing *in situ* zymography in DG and CA1 from a control animal (A, E) and from ischemic animals sacrificed at 7 (B, F), 14 (C, G) and 28 days (D, H) after ischemia. Note the increase of fluorescence signal in the DG and in *strata oriens* and *stratum radiatum* with simultaneous decrease in pyramidal cell layer of the CA1 area across the time of reperfusion. All images subjected to direct comparisons were captured at the same exposure and digital gain settings. Photomicrographs are representative of observations made from six animals per time point. Abbreviations: s.o – *stratum oriens*; s.r. – *stratum radiatum*.

**Figure 6:**
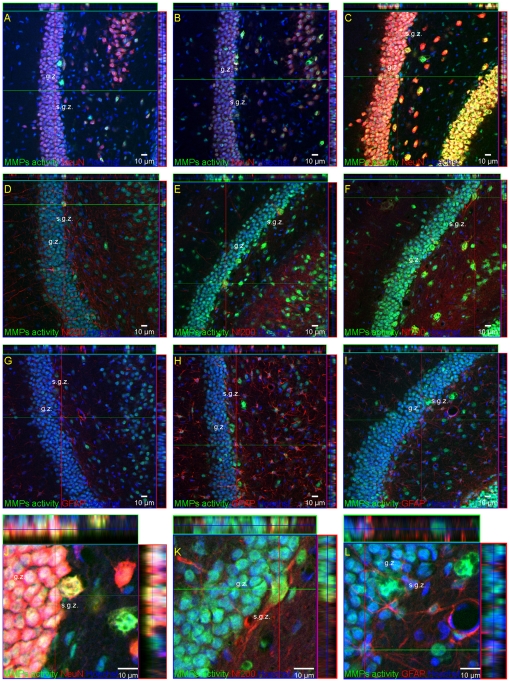
Distribution of metalloproteinases in the DG after global ischemia. Animals were subjected to 5 min of global forebrain ischemia followed by reperfusion. Confocal photomicrographs following co-staining of *in situ* zymography (green) with neuronal markers: NF200 (D, E, F, K) (red) and NeuN (A, B, C, J) (red) or the astrocyte marker GFAP (G, H, I, L) (red) in control ( A, D, G) and at 7 (B, E, H) or 28 days (C, F, I, J, K, L) after ischemia. J, K, L represent magnification (z-stacks)of the picture C,F,I, respectively. Note that MMPs activity was principally associated with neurons. Photomicrographs are representative of observations made from six animals per time point. Scale bar 10 µm Abbreviations: g.z – granular zone; s.g.z- subgranular zone.

In order to test whether changes in net MMP activity accompanied enhanced proliferation and differentiation of stem/progenitor cells in response to ischemia, we used *in situ* zymography in *ex vivo* brain slices, followed by double staining with anti-NF200, NeuN, and GFAP antibodies. We found that in the investigated hippocampal subfields, transient forebrain ischemia affects the activity of MMPs differentially. As depicted in Fig. 5 (A, B, C, D) the gelatinase-associated proteolytic activity in the DG increased progressively across the time of reperfusion. The increment in activity coincides spatially and temporally with the development of stem/progenitor cells. In the neurogenic SGZ the activity of MMPs was seen in NF200-positive neurons as well as in neurons expressing NeuN (Fig. 6). A strong fluorescence signal was observed in Hoechst counterstained neuronal nuclei. However, it was also seen in the cytoplasmic compartment and outside cell bodies. Gelatinolytic activity was also found in the hilus in cells expressing NeuN.

**Figure 7:**
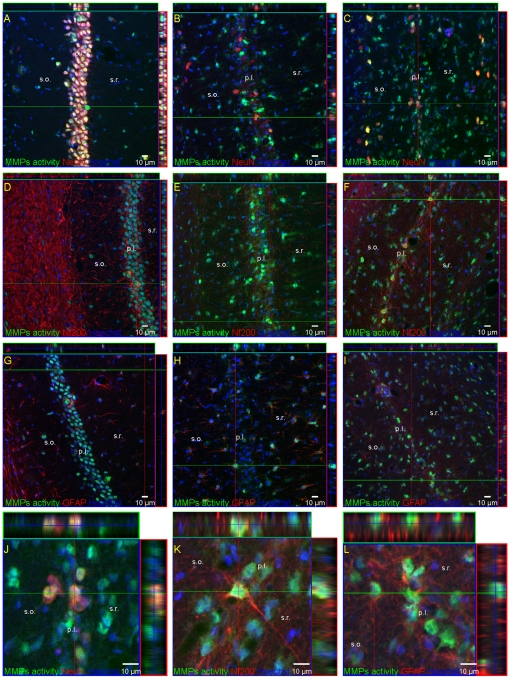
Distribution of metalloproteinases in the CA1 after global ischemia. Confocal photomicrographs following co-staining of *in situ* zymography (green) with neuronal markers: NeuN (A, B, C, J) (red) NF200 (D, E, F, K) (red) or the astrocyte marker GFAP (red) (G, H, I, L) in control (A, D, G) and at 7 (B, E, H) or 28 days (C, F, I, J, K, L) after ischemia. J, K, L represent magnification (z-stacks) of the picture C, F, I respectively. Note the association of MMPs activity with reactive astrocytes GFAP positive in the *stratum oriens* and *stratum radiatum*, probably because of the glial reactivity. Photomicrographs are representative of observations made from six animals per time point. Scale bar 10 µm. Abbreviations: p.l.- pyramidal layer; s.o – *stratum oriens*; s.r. – *stratum radiatum*.

In contrast, in the ischemia-damaged pyramidal cell layer the activity of gelatinases dropped below the control levels during reperfusion (Fig. 5 E, F, G, H). The fluorescence signal in CA1 was detected in neurons expressing NF-200 antigen (Fig. 7). At comparable time points incipient activation of MMPs either associated with or surrounding astrocytes immunoreactive against the GFAP antibody, was found in adjacent brain areas – the *stratum oriens* and *stratum radiatum*. *In situ* zymography in the presence of 1 mM 1,10-O-phenanthroline, a broad spectrum MMP inhibitor, very significantly inhibited the gelatinolytic activity (not shown).

### The effect of MMP inhibitors: SB-3CT, GM6001 and doxycycline on the gelatinolytic activity, growth, proliferation and differentiation of cultured HUCB-NSCs

**Figure 8:**
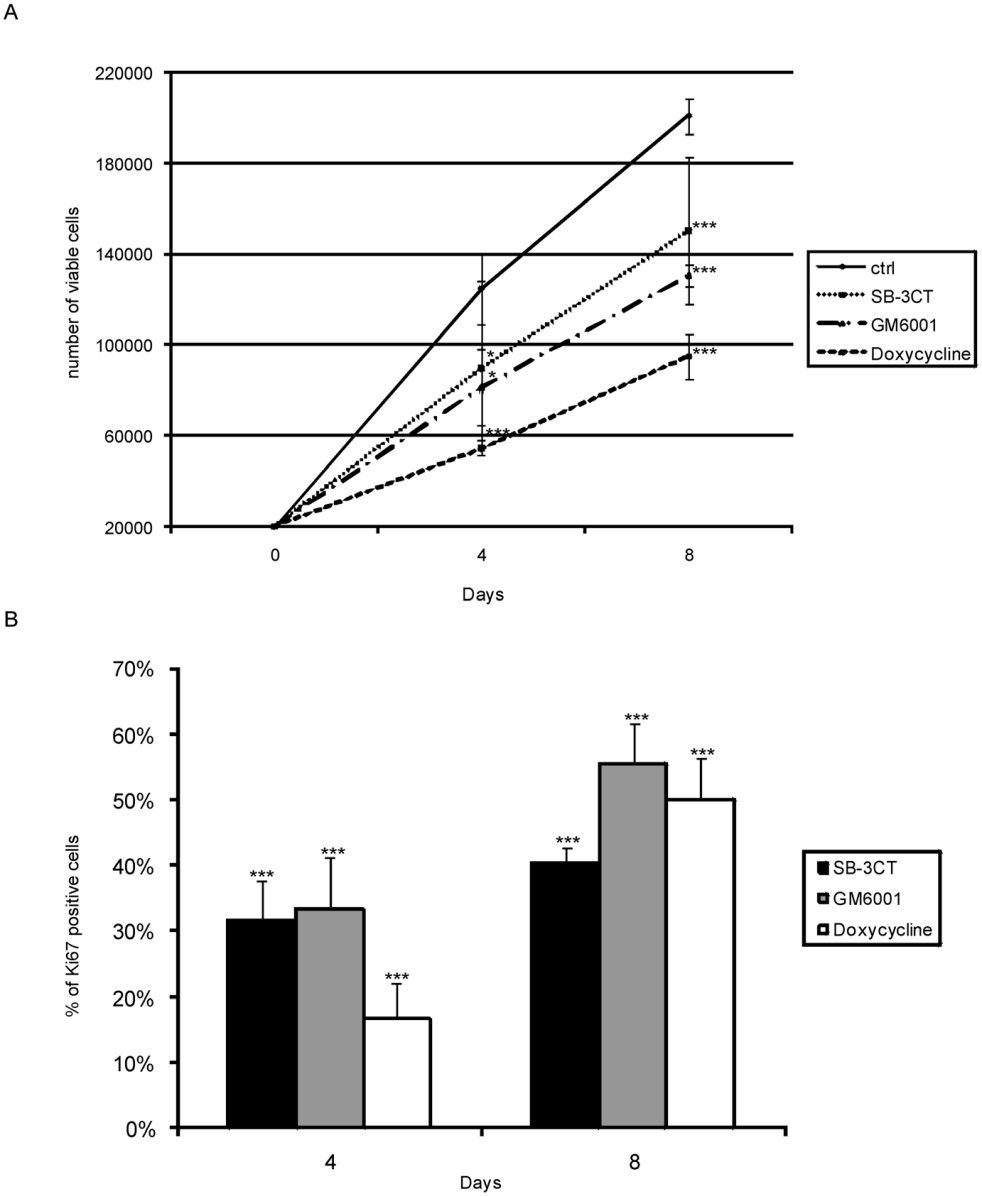
Effect of SB-3CT, GM6001 and doxycycline on the growth and proliferation of HUCB-NSCs. Equivalent numbers of HUCB-NSCs were plated on fibronectin-coated coverslips and grown 8 days in serum-free medium with or without SB-3CT (10 µM), GM6001 (25 µM) or doxycycline (60 µM). A) Graph shows an average number of surviving cells after 4, and 8 days in culture. The addition of SB-3CT, GM6001 or doxycycline to the incubation medium decreased the number of surviving cells. The results (mean values +/− SEM) represent five independent experiments. One-way ANOVA and Bonferroni test: **p<.01; ***p<.001, between treatments (inhibitors vs control value). B) Graph shows the rate of HUCB-NSCs proliferation expressed as a % of control of Ki67- immunopositive cells. The present of SB-3CT, GM6001 or doxycycline in the culture reduced markedly the number of proliferating cells. The results (mean values +/− SEM) represent five independent experiments. One-way ANOVA and Bonferroni test: ***p<.001, between treatments (inhibitors vs control value).

Equivalent numbers of HUCB-NSCs were plated onto fibronectin-coated coverslips and grown in serum-free medium with/or without added pharmacological agents, SB-3CT, GM 6001 or doxycycline. Fig. 8A illustrates the average time-course of growth rate during 8 days in culture. Quantitation of cells in control conditions (without inhibitors) shows continuous increase in the number of viable cells vs initially plated (c.10-fold). The addition of MMPs inhibitor – SB-3CT (10 µM), GM6001 (25 µM) or doxycycline (60 µM) - to the culture medium significantly reduced the viability of HUCB-NSCs at 4 and 8 days in culture. At this time, the number of living cells was reduced by about 30% in the presence of SB-3CT and GM 6001 and 60% by doxycycline, as compared with untreated cells [*F*(3, 52) = 10.42, *p*<.001]. Despite a significantly lower proportion of live to total cells in the treated cultures as compared with the control, living cells show a stable growth rate.

**Figure 9:**
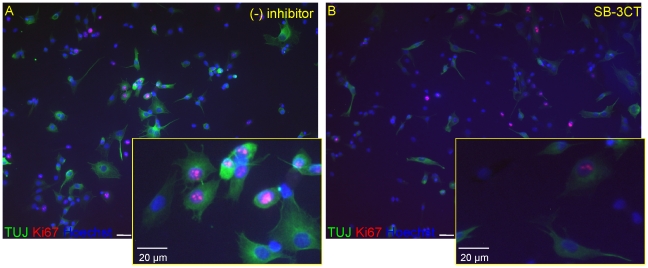
Effect of SB-3CT (10 µM) on the Ki67 and Tuj1 positive cells in HUCB-NSs culture. Equivalent numbers of HUCB-NSCs were plated on fibronectin-coated coverslips and grown 8 days in serum-free medium with or without SB-3CT (10 µM). Cells were stained for Ki67 (red) and Tuj1 (green). The cell nuclei counterstained with Hoechst (blue). Note the decreased number of immunolabeled cells in the presence of SB-3CT. Scale bar 20 µm.

In order to test the influence of the MMP inhibitors on cell proliferation, we used a marker of dividing cells, Ki67. Quantitation of immunopositive cells indicates that HUCB-NSCs proliferate consistently on fibronectin-coated plates. The addition of SB-3CT, GM6001 or doxycycline resulted in a markedly reduced proportion of Ki67-positive cells (in relation to control) - to the average range 20%–30% at 4 and 40–50% at 8 days in culture [*F*(3, 56) = 22.16, *p*<.001] (Figs. 8B and 9).

**Figure 10:**
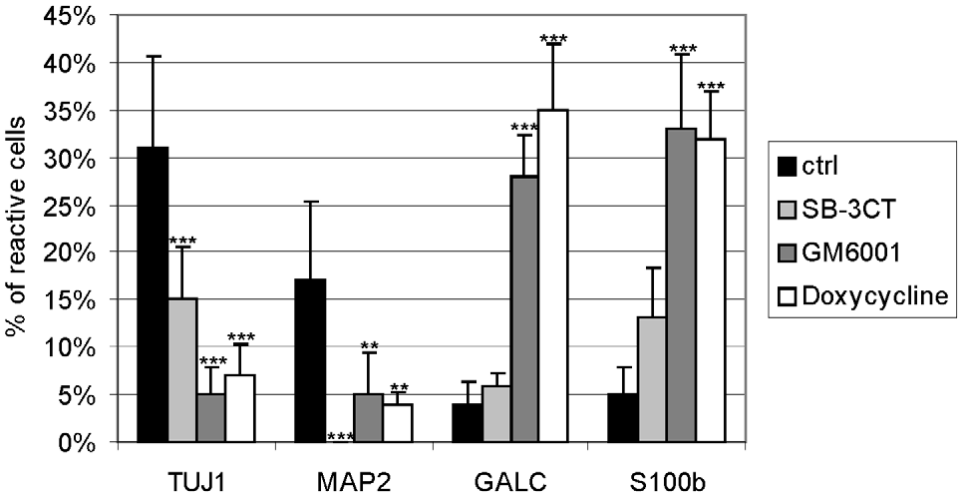
Effects of GM6001 and doxycycline on the differentiation of HUCB-NSCs. Equivalent numbers of HUCB-NSCs were plated on fibronectin-coated coverslips and grown 8 days in serum-free medium with or without SB-3CT (10 µM), GM6001 (25 µM) or doxycycline (60 µM). The graph represents the percentages of immunolabeled cells relative to the total cell population that are present in the culture. Note the decrease of the relative number of cells presenting neuronal antigen and simultaneous increase of the relative amount of glial cells in the presence of SB-3CT, GM6001 and doxycycline. The results (mean values +/− SEM) represent five independent experiments. One-way ANOVA and Bonferroni test: **p<.01, ***p<.001, between treatments (inhibitors vs control value).

**Figure 11:**
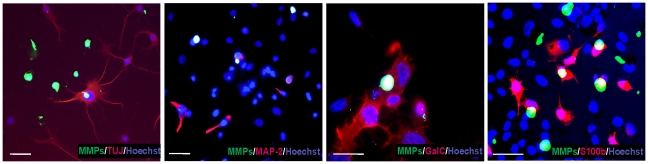
Distribution of metalloproteinases in HUCB-NSCs. Equivalent numbers of HUCB-NSCs were plated on fibronectin-coated coverslips and grown 8 days in culture in serum-free medium. Confocal photomicrographs show co-staining of *in situ* gelatinolytic activity (green) with neuronal markers (red): Tuj1 and MAP2, the astrocytic marker S100beta and oligodendrocytes marker GalC. Double staining appears in yellow. The cell nuclei counterstained with Hoechst (blue). Note the metalloproteinase activity in all tested phenotypes. Photomicrographs are representative of observations made from at least three independent experiments. Scale bar 20 µm.

We next tested whether these inhibitors might affect the differentiation of HUCB-NSCs into neurons and glia. In control cultures at 8 DIV, approximately 32% of the entire cell population was immunopositive for Tuj1 (immature neurons) while 17% were stained for MAP-2 (mature neurons). Analysis of cells immunolabelled for S100beta and GalC showed that astroglia and oligodendroglia accounted for 5% and 4% of cells, respectively (Fig. 10). We also tried to determine which cell phenotypes express endogenous MMPs. High-resolution confocal analysis indicated that MMP activity was localized in all tested cell types: in immature and differentiated neurons (Tuj1- and MAP2^+^, astrocytes (S100beta^+^) and oligodendrocytes (GalC^+^) (Fig. 11). This observation suggests the involvement of metalloproteinases in HUCB-NSCs differentiation. The presence of investigated pharmacological agents altered the profile of differentiation - they inhibited the generation of neurons and promoted the differentiation into oligodendrocytes and astrocytes (Fig. 10). As shown in the graph, among the remaining cells, the percentage of immature neurons (Tuj1-positive) was reduced to 15% in the presence SB-3CT (see also Fig. 9) and to 5% and 7.5% in the cultures treated with GM6001 and doxycycline, respectively [control vs inhibitors *F*(3, 26) = 21.92, *p*<.001]. The addition of MMP inhibitors decreased as well the proportion of mature neurons (MAP-2+), with the most pronounced effect of SB-3CT, which almost completely blocked their generation [control vs inhibitors *F*(3, 18) = 11,73. In the presence of doxycyline and GM6001 *p* = .003, in the presence of SB-3CT *p*<.001].

Concomitantly, the percentage of cells expressing the astroglial and oligodendroglial markers (S100beta and GalC) was higher, and increased by about 6- and 8-fold, respectively, in the presence of GM6001 and doxycycline, as compared with the untreated culture. The number of ofr GalC- or S100beta-positive cells was affected similarly in the presence of both these inhibitors [*F*(2, 14) = 65,41, *p*<.001 and *F*(2, 16) = 32,35, *p*<.001, respectively]. The less effect on the promotion of glial cells differentiation was noted in the case of SB-3CT – the cells expressing GalC increased only 1.5-fold and S100beta about 2.5-fold, versus control.

In contrast, the serine proteinase inhibitor Pefabloc (5 mM) and the furin inhibitor Dec-RVKR-CMK (50 µM) did not alter the profile of differentiation, as compared with control cultures (results not shown).

## Discussion

In agreement with recent studies, our results demonstrate that adult neural progenitors proliferate *in situ* in response to forebrain ischemia. Furthermore, current data also demonstrate that the stimulation of neural stem cell development in the dentate area of the hippocampus after forebrain ischemia is accompanied by a substantial increase in MMPs activity. The timing and magnitude of the elevation of net proteolytic activity after ischemia correlate well with the acceleration of stem/progenitor cells proliferation and further differentiation in the DG, which strongly supports the contribution of MMPs in post-ischemic neurogenesis. Our observations provide a new insight into the role of MMPs in contrast with the detrimental roles traditionally attributed to these enzymes in cerebral ischemia [13], [26], [27], and are in line with the particularly interesting data reported recently by Barkho *et al* [19], supporting a role for endogenous MMPs in stroke-induced neurogenesis.

The analysis of post-ischemic rate of cell birth and differentiation reveals different neurogenic potentials of the hippocampal subfields. In the known neurogenic zone of the DG, cell proliferation increased markedly, compared to control animals. Some of the proliferating BrdU-labeled cells expressed NF-200 as early as 24 h after the injection of BrdU, indicating that some dividing progenitor cells commit to neuronal differentiation. Interestingly, we and others have previously shown MMP activity in the immature neurons [28], [29]. At later time points (14 and 28 days) after ischemic insult numerous progenitor cells relocate into the granular cell layer and become mature granule neurons in line with previous studies showing that NeuN is expressed after SGZ neuronal precursors migrate into the granular cell layer (1,2). The double BrdU-GFAP positive cells found occasionally are probably neural stem cells and may suggest GFAP labeling of non proliferating astroglia present in neurogenic zones [30]. This finding supports previous reports where the proliferation of astroglia in the DG after global ischemia is unremarkable [31], [32].

In contrast, in the damaged CA1 pyramidal layer we noticed a small number of BrdU+ cells, only slightly greater than in the control. Some of these cells exhibited the NF-200 antigen which appeared early in neuronal development, but did not express mature neuronal antigen-NeuN, suggesting they undergo programmed cell death before attaining maturity. Moreover, we found no evidence of SGZ neural stem cells migration into the CA1 to replace neurons lost after ischemia in line with previously reported data showing that proliferating cells in the SGZ travel only to the adjacent GCL [33]. From the above, it follows that in the present experimental conditions, the expected endogenous regenerative capacity fails as a source of meaningful compensation for lost neuronal circuits and that the CA1 area merely displays gliogenesis. These data are in disagreement with previous reports indicating that newly-formed neurons originated in the brain neurogenic zones (SVZ or SGZ), have the capacity to migrate into the injured CA1 area to integrate the existing brain circuitry [5], [6]. The reasons for such a discrepancy may be due to differences in experimental protocols.

It is worth noting that the vast majority of proliferating BrdU+cells were present in the *stratum oriens* and *stratum radiatum* of the CA1, rather than in the pyramidal cell layer. These cells exhibited characteristic features of astroglial phenotype: flat spread or stellate cells with filamentous GFAP staining.

A clear picture of the factors responsible for neurogenesis in the SGZ is still elusive. Several findings clearly support that ischemia-induced neuronal progenitor development is certainly controlled by the cooperative action of many intrinsic and extrinsic factors, which may contribute to the difference in SGZ/CA1 neurogenic potential. One of the most interesting findings obtained in the current work is that ischemia elicits contrasting effects on the spatial pattern of net MMP activity that matches the progression of proliferation in the DG across time and correlates well with the process of differentiation of stem/progenitor cells into mature neurons. Such a spatio-temporal relationship between activation of MMPs and neurogenesis may suggest a casual link between these processes. This finds strong support in our cell culture experiments showing that the SB-3CT mediated inhibition of endogenous MMP activity, (particularly MMP-2 and MMP-9), of HUCB-NSCs significantly reduced both their proliferation and their differentiation toward the neuronal lineage. However, we cannot exclude that the reduced number of immature and mature neurons (Tuj1- and MAP-2 positive, respectively) might likely be related to a lower level of generation thereof, and/or their decreased survival. Simultaneously, the number of oligodendrocytes and astrocytes augmented compared to the control, probably due to increased proliferation. These data support our *in vivo* results relative to the involvement of MMPs in the development of progenitors. They also provide an intriguing model in which MMPs could modulate the nuclear regulated functions of HUCB-NSCs. Further support to stress the importance of MMPs in neurogenesis as compared with other proteinases stems from the failure of serine proteinase and furin inhibitors (Pefabloc and Dec-RVKR-CMK) to modulate this process. Consistent with this notion, there remain emerging *in vitro* and *in vivo* data pointing to regulatory roles of MMPs in neuroblast migration across tissue matrices [18], [34] as well as during the proliferation and differentiation of neural precursor cells after global and focal ischemia in rodents [17], [19]. On the other hand, partial preservation of HUCB-NSCs proliferation in the presence of metalloproteinase inhibitor suggests that the process is not entirely dependent on MMP activity.

It is not possible at present to define precisely which of their pleiotropic functions of MMPs are directly linked to post-ischemic neurogenesis. One likely scenario would involve proteolytic modulation of guidance molecules and/or the remodeling of the ECM [35]. The latter may uncover cryptic sites or liberate soluble fragments [36], [37] that promote migration. It is therefore plausible that the relocation of cells from the SGZ to the GCL in the DG observed in our study may be facilitated by MMP-mediated breakdown of ECM barriers impeding cell movement. Complementary to their role in cell migration, MMPs could also promote neurite extension of newly integrated progenitor cells as suggested by recent findings involving MMP-2 and MMP-3 in dendro-axonal growth of cortical immature neurons [38], [39].

MMP-mediated conversion of several trophic factors to their biologically active forms may also produce signals supporting neurogenesis [40].

One of the interesting results from this study is the presence of MMPs activity in neuronal nuclei in the DG area, consistent with recent findings showing gelatinolytic activity in the neuronal nuclei of ischemic brain as well as in the nuclei of cultured astrocytes [41]–[43]. Nuclear metalloproteinase activity may influence the mechanisms by which neural stem/progenitor cells adjust their gene expression program but no information is available at present. Clearly, further investigation will be necessary to confirm this hypothesis. Nevertheless, regardless of the mechanism of MMPs action in the DG, the spatial and temporal relationship between the activation of MMPs and accelerated BrdU incorporation argues strongly for their involvement in ischemia-induced neurogenesis.

Why neurogenesis fails in the post-ischemic CA1 pyramidal cell layer is currently unclear. This may obey to different events that challenge tissue restoration including substantial ECM deposition and glial scar formation [44], [45], along with high levels of at least one inhibitor of metalloproteinases (TIMP-1) found in reactive astrocytes after global ischemia [12]. The latter may contribute to the low MMP activity observed in this area and favor altogether the formation of a glial scar that hampers the remodeling of the damaged tissue required for its regeneration. The high level of invading microglial cells usually found in the early stages of the ischemic lesion may also contribute to disturb the homeostasis of the extracellular environment necessary for progenitors to proliferate and differentiate. In this regard, it has been proposed that early microglial expression of MMP-9 in the CA1 of rats after global ischemia, contributes to early neurodegeneration in this area [12].

Unlike in the pyramidal cell layer significant activation of gelatinase-associated activity was observed in the *stratum oriens* and *stratum radiatum* at some distance from the injured site. As we have reported previously, ischemia-induced changes are located in the dendritic region of CA1 pyramidal neurons of the *stratum radiatum* and then spread out to the *stratum oriens* [46]. In these regions the stress response of postinjured tissue seems to be associated with the appearance of reactive astrocytes and increased MMP activity that may facilitate delayed astrocyte-mediated tissue remodeling and repair at the periphery of the lesion.

### Conclusions

The spatio-temporal relationship between neurogenic-associated processes and gelatinase activity observed after forebrain ischemia in the *dentate gyrus* of the adult rodent brain may indicate that the metalloproteinases are among the discussed mechanism(s) which govern the development of stem/progenitor cells. The importance of MMPs in neurogenesis finds strong support in our in vitro study showing inhibition of proliferation and differentiation in the presence of metalloproteinases inhibitors.

## References

[1] Liu J, Solway K, Messing RO, Sharp FR (1998). Increased neurogenesis in the dentate gyrus after transient global ischemia in gerbils.. J Neurosci.

[2] Jin K, Minami M, Lan JQ, Mao XO, Batteur S (2001). Neurogenesis in the dentate subgranular zone and rostral subventricular zone after focal cerebral ischemia in the rat.. Proc Natl Acad Sci USA.

[3] Parent JM, Vexler ZS, Gong C, Derugin N, Ferriero DM (2002). Rat forebrain neurogenesis and striatal neuronal replacement after focal stroke.. Ann Neurol.

[4] Burns TC, Varfaillie CM, Low WC (2009). Stem cells for ischemic brain injury: A critical review.. J Comp Neurol.

[5] Nakatomi H, Kuriu T, Okabe S, Yamamoto S, Hatano O (2002). Regeneration of hippocampal pyramidal neurons after ischemic brain injury by recruitment of endogenous neural progenitors.. Cell.

[6] Bendel O, Bueters T, Euler M, Ogren SO, Sandin J (2005). Reappearance of hippocampal CA1 neurons after ischemia is associated with recovery of learning and memory.. J Cereb Blood Flow Metab.

[7] Bovetti S, Bovolin P, Perroteau I, Puche AC (2007). Subventricular zone-derived neuroblast migration to the olfactory bulb is modulated by matrix remodeling.. Eur J Neurosci.

[8] Ethel IM, Ethel DW (2007). Matrix metalloproteinases in brain development and remodeling: Synaptic functions and targets.. J Neurosci Res.

[9] Rivera S, Khrestchatisky M, Kaczmarek L, Rosenberg GA, Jaworski DM (2010). Metzincin Proteases and their Inhibitors, Foes or Friends in Nervous System Physiology?. J Neurosci.

[10] Dzwonek J, Rylski M, Kaczmarek L (2004). Matrix metalloproteinases and their endogenous inhibitors in neuronal physiology of the adult brain.. FEBS Lett.

[11] Yong VW (2005). Metalloproteinases mediators of pathology and regeneration in the CNS.. Nat Rev Neurosci.

[12] Rivera S, Ogier C, Jourquin J, Timsit S, Szklarczyk AW (2002). Gelatinase B and TIMP-1 are regulated in a cell- and time- dependent manner in association with neuronal death and glial reactivity after global forebrain ischemia.. Eur J Neurosci.

[13] Gasche Y, Soccal PM, Kanemitsu M, Copin J-C (2006). Matrix metalloproteinases and diseases of the central nervous system with a special emphasis on ischemic brain.. Front Biosci.

[14] Frolichsthal-Schoeller P, Vescovi AL, Krekoski CA, Murphy G, Edwards DR (1999). Expression and modulation of matrix metalloproteinase-2 and tissue inhibitors of metalloproteinases in human embryonic CNS stem cells.. NeuroReport.

[15] Mannello F, Tonti GAM, Bagnara GP, Papa S (2006). Role and function of matrix metalloproteinases in the differentiation and biological characterization of mesenchymal stem cell.. Stem Cells.

[16] Morris DC, Zhang ZG, Zhang R, LeTourmeau Y, Gregg SR (2006). Stroke increases expression of matrix metalloproteinases and p21–activated protein kinase in neural progenitor cells.. Acad Emerg Med.

[17] Lu L, Tonchev AB, Kaplamadzhiev DB, Boneva NB, Mori Y (2008). Expression of Matrix Metalloproteinases in the Neurogenic Niche of the Adult Monkey Hippocampus after Ischemia.. Hippocampus.

[18] Lee SR, Kim H-J, Rogowska J, Zhao BQ, Bhide P (2006). Involvement of Matrix Metalloproteinase in Neuroblast Cell Migration from the Subventricular Zone after Stroke.. J Neurosci.

[19] Barkho BZ, Munoz AE, Li X, Li L, Cunningham LA (2008). Endogenous Matrix Metalloproteinase MMP-3 and MMP-9 Promote Differentiation and Migration of Adult Neural Progenitor Cells in Response to Chemokines.. Stem Cells.

[20] Kang SS, Kook JH, Hwang S, Park SH, Nam SC (2008). Inhibition of matrix metalloproteinase-9 attenuated neural progenitor cell migration after photothrombotic ischemia.. Brain Res.

[21] Wojcik L, Sawicka A, Rivera S, Zalewska T (2009). Neurogenesis in gerbil hippocampus following brain ischemia: focus on the involvement of metalloproteinases.. Acta Neurobiol Exp.

[22] Domanska-Janik K, Bong P, Bronisz-Kowalczyk A, Zajac H, Zabłocka B (1999). AP1 transcriptional factor activation and its relation to apoptosis of hippocampal CA1 pyramidal neurons after transient ischemia in gerbils.. J Neurosci Res.

[23] Ogier C, Bernard A, Chollet AM, Le Diguardher T, Hanessian S (2006). Matrix metalloproteinase -2 (MMP-2) regulates astrocyte motility in connection with the actin cytoskeleton and integrins.. Glia.

[24] Buzanska L, Jurga M, Stachowiak EK, Stachowiak MK, Domanska-Janik K (2006). Neural stem-like cell line derived froma nonhematopoietic population of human umbilical cord blood.. Stem Cells Dev.

[25] Szymczak P, Sypecka J, Zalewska T (2009). Relationship between extracellular matrix components and MMPs activity during development of neural stem cells from umbilical cord blood (HUCB-NSC).. (Abstract) Acta Neurobiol Exp.

[26] Asahi M, Asahi K, Jung JC, del Zoppo GJ, Fini ME (2000). Role for matrix metalloproteinase 9 after focal cerebral ischemia: effects of gene knockout and enzyme inhibition with BB-94.. J Cereb Blood Flow Metab.

[27] Zalewska T, Ziemka-Nalecz M, Sarnowska A, Domanska-Janik K (2002). Involvement of MMPs in delayed neuronal death after global ischemia.. Acta Neurobiol Exp.

[28] Lee CZ, Xu B, Hashimoto T, Yang GY, Young WL (2004). Doxycycline suppresses cerebral matrix metalloproteinase-9 and angiogenesis induced by focal hyperstimulation of vascular endothelial growth factor in a mouse model.. Stroke.

[29] Jablonska A, Wojcik L, Zalewska T, Domanska-Janik K, Lukomska B (2009).

[30] Wurmser AE, Palmer TD, Gage FH (2004). Cellular interactions in the stem cell niche.. Science.

[31] Kato H, Takahashi A, Itoyama Y (2003). Cell cycle protein expression in proliferating microglia and astrocytes following transient global cerebral ischemia in the rat.. Brain Res Bull.

[32] Tonchev AB, Yamashima T, Zhao L, Okano HJ, Okano H (2003). Proliferation of neural and neuronal progenitors after global brain ischemia in young adult macaque monkeys.. Mol Cell Neurosci.

[33] Jin K, Sun Y, Xie L, Peel A, Mao XO (2003). Directed migration of neuronal precursors into the ischemic cerebral cortex and striatum.. Mol Cell Neurosci.

[34] Tsukatani T, Fillmore HL, Hamilton HR, Holbrook EH, Costanzo RM (2003). Matrix metalloproteinase expression in the olfactory epithelium.. Neuro Report.

[35] Nagase H, Woessner JF (1999). Matrix metalloproteinases.. J Biol Chem.

[36] Gianelli G, Falk-Marzillier J, Schiraldi O, Stetler-Stevenson WG, Quaranta V (1997). Induction of cell migration by matrix metalloprotease-2 cleavage of laminin-5.. Science.

[37] Xu J, Rodriguez D, Petitclerc E, Kim JJ, Hangai M (2001). Proteolytic exposure of a cryptic site within collagen type IV is required for angiogenesis and tumor growth in vivo.. J Cell Biol.

[38] Gonthier B, Koncina E, Satkauskas S, Perraut M, Roussel G (2009). A PKC-dependent recruitment of MMP-2 controls semaphorin-3A growth-promoting effect in cortical dendrites.. PLoS One.

[39] Ould-yahoui A, Tremblay E, Sbai O, Ferhat L, Bernard A (2009). A new role for TIMP-1 in modulating neurite outgrowth and morphology of cortical neurons.. PLoS One.

[40] Bruno MA, Cuello AC (2006). Activity-dependent release of precursor nerve growth factor, conversion to mature nerve growth factor, and its degradation by protease cascade.. Proc Natl Acad Sci USA.

[41] Amantea D, Corasaniti MT, Mercuri NB, Bernardi G, Bagetta G (2007). Brain regional and cellular localization of gelatinase activity in rat that have undergone transient middle cerebral artery occlusion.. Neuroscience.

[42] Yang Y, Candelario-Jalil E, Thompson JF, Cuadrado E, Estrada EY (2010). Increased intranuclear metalloproteinase activity in neurons interferes with oxidative DNA repair in focal cerebral ischemia.. J Neurochem.

[43] Sbai O, Ould-Yahoui A, Ferhalt L, Gueye Y, Bernard A (2010). Differential vesicular distribution and trafficking of MMP-2, MMP-9, and their inhibitors in astrocytes.. Glia.

[44] Fawcett JW, Asher RA (1999). The glial scar and central nervous system repair.. Brain Res Bull.

[45] Nedergaard M, Dirmagl U (2005). Role of glial cells in cerebral ischemia.. Glia.

[46] Ziemka-Nalecz M, Zalewska T, Zajac H, Domanska-Janik K (2003). Decrease of PKC precedes other cellular signs of calpain activation after transient cerebral ischemia.. Neurochem Int.

